# Crystal structure of 6,9-dimethyl-7*H*-[1,2,4]triazolo[4,3-*b*][1,2,4]triazepin-8(9*H*)-one 0.40-hydrate

**DOI:** 10.1107/S2056989014025687

**Published:** 2015-01-01

**Authors:** Abdellah Harmaoui, Rachid Bouhfid, El Mokhtar Essassi, Mohamed Saadi, Lahcen El Ammari

**Affiliations:** a Laboratoire de Chimie Organique Hétérocyclique URAC 21, Pôle de Compétences Pharmacochimie, Av. Ibn Battouta, BP 1014, Faculté des Sciences, Université Mohammed V, Rabat, Morocco; b Moroccan Foundation for Advanced Science, Innovation and Research (MASCIR), Rabat, Morocco; c Laboratoire de Chimie du Solide Appliquée, Faculté des Sciences, Université Mohammed V, Avenue Ibn Battouta, BP 1014, Rabat, Morocco

**Keywords:** crystal structure, 1,2,4-triazepin-8(9*H*)-one, pharmacological and biological activities, hydrogen bonding

## Abstract

In the mol­ecule of the title compound, C_7_H_9_N_5_O·0.40H_2_O, the seven-membered heterocyclic ring exhibits a boat conformation, whereas the five-membered triazole ring is almost planar (r.m.s. deviation = 0.005 Å). In the crystal, centrosymmetric dimers are linked by pairs of C—H⋯O hydrogen bonds into dimers, which are further connected *via* O—H⋯N and C—H⋯N hydrogen bonds, forming a three-dimensional network. The structure contains a partially occupied water mol­ecule lying on a twofold axis with an occupancy factor of 0.4.

## Related literature   

For pharmacological and biological activities of 1,2,4-triazole and 1,2,4-triazepine derivatives, see: Gupta *et al.* (2011 ▸); Mathew *et al.* (2006 ▸); Reed *et al.* (2010 ▸). For related structures, see: Essassi *et al.* (1977 ▸); Doubia *et al.* (2007 ▸); Zemama *et al.* (2009 ▸).
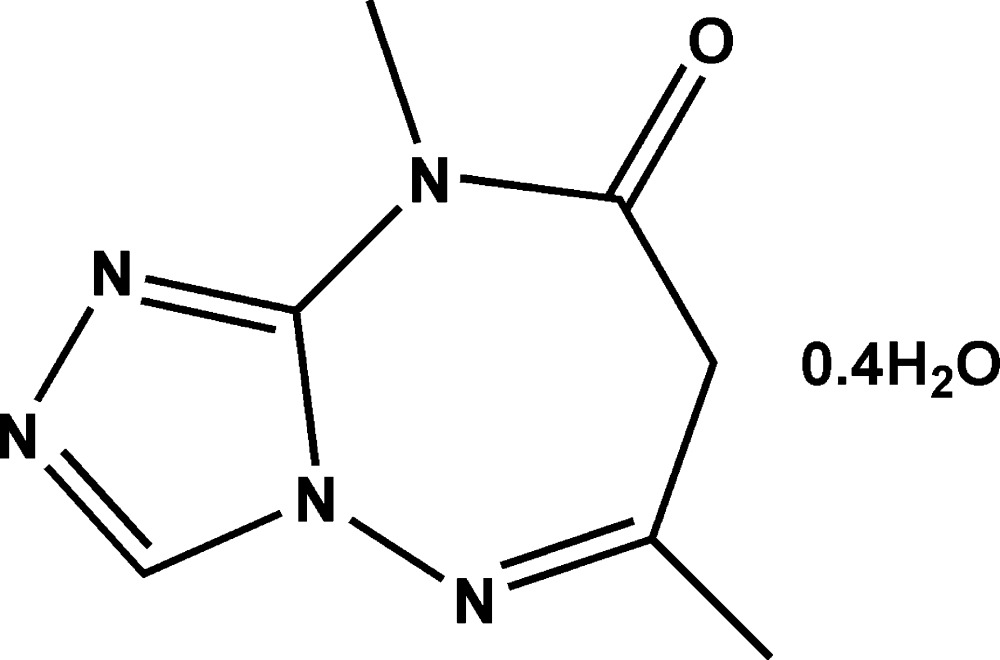


## Experimental   

### Crystal data  

C_7_H_9_N_5_O·0.4H_2_O
*M* *_r_* = 186.44
Monoclinic, 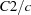

*a* = 11.4970 (18) Å
*b* = 11.4527 (18) Å
*c* = 14.867 (2) Å
β = 109.615 (4)°
*V* = 1843.9 (5) Å^3^
*Z* = 8
Mo *K*α radiation
μ = 0.10 mm^−1^
*T* = 296 K
0.40 × 0.34 × 0.30 mm

### Data collection  

Bruker X8 APEX diffractometer
Absorption correction: multi-scan (*SADABS*; Bruker, 2009 ▸) *T*_min_ = 0.637, *T*_max_ = 0.746
14175 measured reflections
2039 independent reflections
1600 reflections with *I* > 2σ(*I*)
*R*_int_ = 0.033

### Refinement  

*R*[*F* ^2^ > 2σ(*F* ^2^)] = 0.042
*wR*(*F* ^2^) = 0.125
*S* = 1.04
2039 reflections
123 parameters
H-atom parameters constrained
Δρ_max_ = 0.28 e Å^−3^
Δρ_min_ = −0.20 e Å^−3^

### 

Data collection: *APEX2* (Bruker, 2009 ▸); cell refinement: *SAINT-Plus* (Bruker, 2009 ▸); data reduction: *SAINT-Plus*; program(s) used to solve structure: *SHELXS97* (Sheldrick, 2008 ▸); program(s) used to refine structure: *SHELXL97* (Sheldrick, 2008 ▸); molecular graphics: *ORTEP-3 for Windows* (Farrugia, 2012 ▸); software used to prepare material for publication: *PLATON* (Spek, 2009 ▸) and *publCIF* (Westrip,2010 ▸).

## Supplementary Material

Crystal structure: contains datablock(s) I. DOI: 10.1107/S2056989014025687/rz5141sup1.cif

Structure factors: contains datablock(s) I. DOI: 10.1107/S2056989014025687/rz5141Isup2.hkl

Supporting information file. DOI: 10.1107/S2056989014025687/rz5141Isup3.cml

**Fig. 1.**
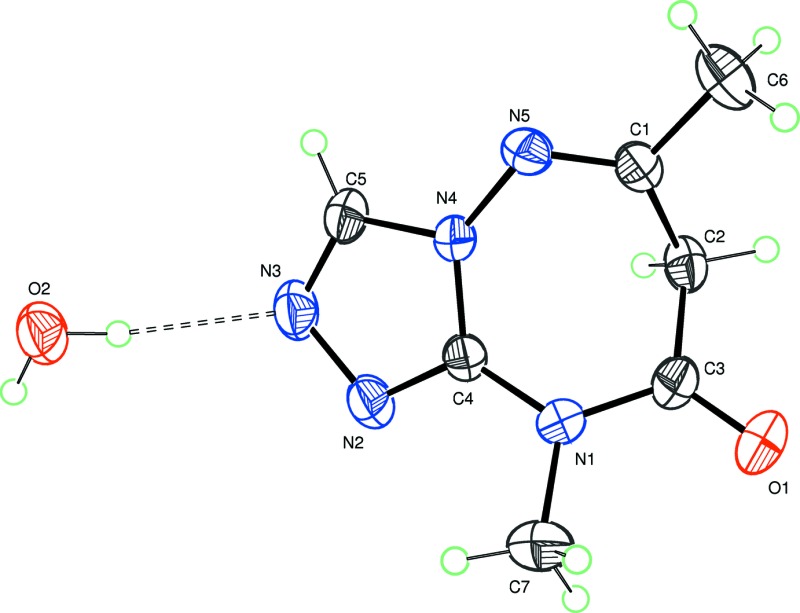
The asymmetric unit of the title compound with displacement ellipsoids drawn at the 50% probability level, showing the intermolecular O—H···N hydrogen bond (dashed line).

. DOI: 10.1107/S2056989014025687/rz5141fig1.tif
The asymmetric unit of the title compound with displacement ellipsoids drawn at the 50% probability level, showing the inter­molecular O—H⋯N hydrogen bond (dashed line).

**Fig. 2.**
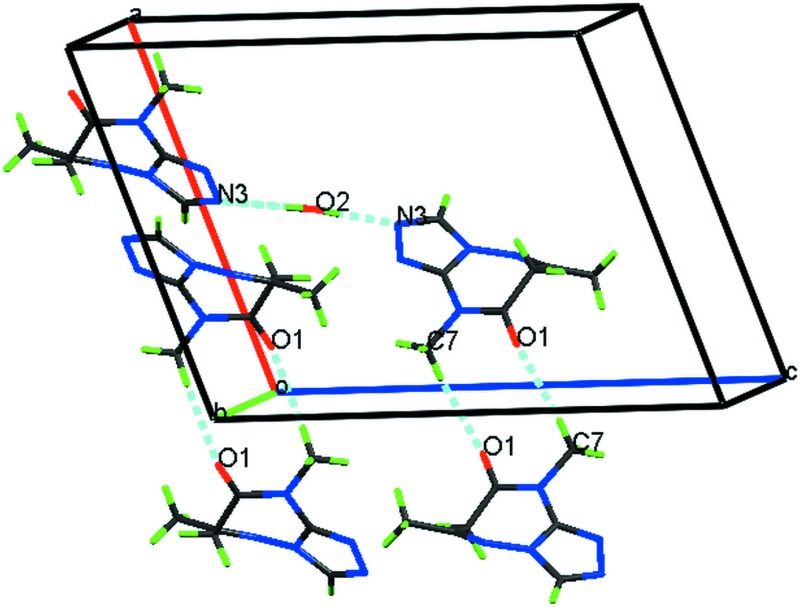
Partiel crystal packing of the title compound, showing molecules linked through C—H···O and O—H···N hydrogen bonds (dashed lines).

. DOI: 10.1107/S2056989014025687/rz5141fig2.tif
Partiel crystal packing of the title compound, showing mol­ecules linked through C—H⋯O and O—H⋯N hydrogen bonds (dashed lines).

CCDC reference: 1035668

Additional supporting information:  crystallographic information; 3D view; checkCIF report

## Figures and Tables

**Table 1:** Hydrogen-bond geometry (, )

*D*H*A*	*D*H	H*A*	*D* *A*	*D*H*A*
C2H2*A*N3^i^	0.97	2.58	3.449(3)	149
C5H5O1^ii^	0.93	2.29	3.211(2)	173
O2H1N3^iii^	0.87	2.08	2.939(2)	167

Symmetry codes: (i) 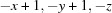; (ii) 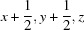; (iii) 
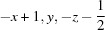
.

## supplementary crystallographic information

### S1. Comment

1,2,4-Triazole derivatives are known to possess wide biological significance and diverse pharmacological activities (Mathew *et al.*, 2006; Reed *et al.*, 2010). 1,2,4-Triazepine derivatives were also reported to possess antibacterial, antiviral and psychotropic activities (Gupta *et al.*, 2011). They are also the reactants for the synthesis of other heterocyclic compounds (Essassi *et al.*, 1977; Doubia *et al.*, 2007; Zemama *et al.*, 2009). The aim of the present paper is to report the crystal structure of the title compound.

The molecule of the title compound is build up from two fused five- and seven-membered rings linked to two methyl groups and crystallizing with a partial water molecule as shown in Fig. 1. The triazepine ring adopts a boat conformation as indicated by the puckering amplitude *Q* = 0.7865 (17) Å and spherical polar angle θ = 88.80 (12)°, with φ = 60.07 (13)°. The triazole ring is close to be planar, with a maximum deviation of 0.007 (2) Å for atom C5. In the crystal, centrosymmetrically-related molecules are linked by pairs of weak C—H···O hydrogen bonds into dimeric units, which are further connected into a three-dimensional network by O—H···N and C—H···O hydrogen bonds (Fig. 2, Table 1).

### S2. Experimental

To a solution of 1 g (0,06 mol) of6-methyl-7*H*-[1,2,4]triazolo[4,3-*b*][1,2,4]triazepin-8(9*H*)-one in 30 ml of sodium methoxide (prepared from 30 ml of methanol and 0.15 g of sodium) was added 1 g (0.007 mol) of methyl iodide and the mixture was heated for 5 h. The solution was then concentrated to dryness under reduced pressure and the residue was extracted with chloroform. The precipitate obtained was chromatographed on a silica column (eluent: chloroform/ethanol 95:5 *v*/*v*). The purified product was crystallized from ethanol to give colourless crystals with a yield of 50%.

### S3. Refinement

All H atoms were located in a difference Fourier map and refineded as riding, with C—H = 0.93-0.97 Å, O–H = 0.90 Å, and with *U*_iso_(H) = 1.2 *U*_eq_(C) or 1.5 *U*_eq_(C, O) for methyl and water H atoms. The oxygen atom of the water molecule lies on a two-fold axis with an occupancy factor of 0.4.

### Crystal data

**Table:**

C_7_H_9_N_5_O·0.4H_2_O	*F*(000) = 784
*M**_r_* = 186.44	*D*_x_ = 1.343 Mg m^−^^3^
Monoclinic, *C*2/*c*	Mo *K*α radiation, λ = 0.71073 Å
Hall symbol: -C 2yc	Cell parameters from 2039 reflections
*a* = 11.4970 (18) Å	θ = 2.6–27.1°
*b* = 11.4527 (18) Å	µ = 0.10 mm^−^^1^
*c* = 14.867 (2) Å	*T* = 296 K
β = 109.615 (4)°	Block, colourless
*V* = 1843.9 (5) Å^3^	0.40 × 0.34 × 0.30 mm
*Z* = 8	

### Data collection

**Table:**

Bruker X8 APEX diffractometer	2039 independent reflections
Radiation source: fine-focus sealed tube	1600 reflections with *I* > 2σ(*I*)
Graphite monochromator	*R*_int_ = 0.033
φ and ω scans	θ_max_ = 27.1°, θ_min_ = 2.6°
Absorption correction: multi-scan (*SADABS*; Bruker, 2009)	*h* = −14→14
*T*_min_ = 0.637, *T*_max_ = 0.746	*k* = −14→14
14175 measured reflections	*l* = −19→19

### Refinement

**Table:**

Refinement on *F*^2^	Primary atom site location: structure-invariant direct methods
Least-squares matrix: full	Secondary atom site location: difference Fourier map
*R*[*F*^2^ > 2σ(*F*^2^)] = 0.042	Hydrogen site location: inferred from neighbouring sites
*wR*(*F*^2^) = 0.125	H-atom parameters constrained
*S* = 1.04	*w* = 1/[σ^2^(*F*_o_^2^) + (0.0565*P*)^2^ + 1.2827*P*] where *P* = (*F*_o_^2^ + 2*F*_c_^2^)/3
2039 reflections	(Δ/σ)_max_ < 0.001
123 parameters	Δρ_max_ = 0.28 e Å^−^^3^
0 restraints	Δρ_min_ = −0.20 e Å^−^^3^

### Special details

**Table:**

Geometry. All e.s.d.'s (except the e.s.d. in the dihedral angle between two l.s. planes) are estimated using the full covariance matrix. The cell e.s.d.'s are taken into account individually in the estimation of e.s.d.'s in distances, angles and torsion angles; correlations between e.s.d.'s in cell parameters are only used when they are defined by crystal symmetry. An approximate (isotropic) treatment of cell e.s.d.'s is used for estimating e.s.d.'s involving l.s. planes.
Refinement. Refinement of *F*^2^ against all reflections. The weighted *R*-factor *wR* and goodness of fit *S* are based on *F*^2^, conventional *R*-factors *R* are based on *F*, with *F* set to zero for negative *F*^2^. The threshold expression of *F*^2^ > σ(*F*^2^) is used only for calculating *R*-factors(gt) *etc*. and is not relevant to the choice of reflections for refinement. *R*-factors based on *F*^2^ are statistically about twice as large as those based on *F*, and *R*- factors based on all data will be even larger.

### Fractional atomic coordinates and isotropic or equivalent isotropic displacement parameters (Å^2^)

**Table:**

	*x*	*y*	*z*	*U*_iso_*/*U*_eq_	Occ. (<1)
C1	0.33958 (13)	0.65257 (14)	0.16698 (10)	0.0369 (3)	
C2	0.34033 (16)	0.52181 (14)	0.16066 (11)	0.0433 (4)	
H2A	0.4176	0.4962	0.1544	0.052*	
H2B	0.3344	0.4885	0.2190	0.052*	
C3	0.23480 (17)	0.47878 (14)	0.07690 (12)	0.0476 (4)	
C4	0.33342 (14)	0.57338 (13)	−0.02393 (10)	0.0362 (3)	
C5	0.46928 (15)	0.70820 (15)	−0.00967 (12)	0.0453 (4)	
H5	0.5204	0.7725	0.0124	0.054*	
C6	0.30729 (19)	0.70739 (19)	0.24624 (12)	0.0571 (5)	
H6A	0.3102	0.7908	0.2412	0.086*	
H6B	0.3652	0.6828	0.3063	0.086*	
H6C	0.2256	0.6839	0.2424	0.086*	
C7	0.1424 (2)	0.4639 (2)	−0.09733 (15)	0.0758 (7)	
H7A	0.1563	0.4934	−0.1533	0.114*	
H7B	0.0623	0.4881	−0.0976	0.114*	
H7C	0.1464	0.3802	−0.0970	0.114*	
N1	0.23754 (13)	0.50997 (12)	−0.01151 (9)	0.0445 (4)	
N2	0.37481 (14)	0.56109 (13)	−0.09512 (10)	0.0478 (4)	
N3	0.46286 (14)	0.64862 (14)	−0.08511 (10)	0.0508 (4)	
N4	0.39103 (11)	0.66406 (11)	0.03305 (8)	0.0346 (3)	
N5	0.36603 (12)	0.72045 (11)	0.10833 (9)	0.0388 (3)	
O1	0.15060 (15)	0.42096 (14)	0.08579 (11)	0.0790 (5)	
O2	0.5000	0.7639 (2)	−0.2500	0.0618 (6)	0.80
H1	0.5068	0.7196	−0.2959	0.093*	0.80

### Atomic displacement parameters (Å^2^)

**Table:**

	*U*^11^	*U*^22^	*U*^33^	*U*^12^	*U*^13^	*U*^23^
C1	0.0326 (7)	0.0460 (8)	0.0320 (7)	0.0009 (6)	0.0109 (6)	−0.0009 (6)
C2	0.0514 (9)	0.0455 (9)	0.0367 (8)	0.0024 (7)	0.0195 (7)	0.0089 (7)
C3	0.0595 (10)	0.0394 (9)	0.0497 (9)	−0.0114 (7)	0.0260 (8)	0.0005 (7)
C4	0.0434 (8)	0.0345 (7)	0.0327 (7)	−0.0018 (6)	0.0153 (6)	0.0007 (6)
C5	0.0470 (9)	0.0483 (9)	0.0455 (9)	−0.0091 (7)	0.0221 (7)	0.0049 (7)
C6	0.0644 (11)	0.0709 (12)	0.0426 (9)	0.0104 (10)	0.0265 (9)	−0.0051 (9)
C7	0.0784 (15)	0.0916 (16)	0.0559 (12)	−0.0408 (13)	0.0204 (11)	−0.0244 (11)
N1	0.0507 (8)	0.0441 (7)	0.0390 (7)	−0.0160 (6)	0.0157 (6)	−0.0057 (6)
N2	0.0624 (9)	0.0490 (8)	0.0380 (7)	−0.0019 (7)	0.0247 (7)	−0.0020 (6)
N3	0.0572 (9)	0.0588 (9)	0.0451 (8)	−0.0025 (7)	0.0287 (7)	0.0055 (7)
N4	0.0393 (7)	0.0348 (6)	0.0332 (6)	−0.0042 (5)	0.0166 (5)	−0.0007 (5)
N5	0.0427 (7)	0.0383 (7)	0.0369 (6)	−0.0023 (5)	0.0154 (5)	−0.0066 (5)
O1	0.0913 (11)	0.0827 (11)	0.0718 (10)	−0.0466 (9)	0.0389 (9)	−0.0012 (8)
O2	0.0912 (18)	0.0554 (13)	0.0507 (13)	0.000	0.0395 (13)	0.000

### Geometric parameters (Å, º)

**Table:**

C1—N5	1.2788 (19)	C5—N4	1.3610 (19)
C1—C6	1.488 (2)	C5—H5	0.9300
C1—C2	1.501 (2)	C6—H6A	0.9600
C2—C3	1.500 (2)	C6—H6B	0.9600
C2—H2A	0.9700	C6—H6C	0.9600
C2—H2B	0.9700	C7—N1	1.472 (2)
C3—O1	1.216 (2)	C7—H7A	0.9600
C3—N1	1.373 (2)	C7—H7B	0.9600
C4—N2	1.306 (2)	C7—H7C	0.9600
C4—N4	1.3632 (19)	N2—N3	1.397 (2)
C4—N1	1.383 (2)	N4—N5	1.4029 (17)
C5—N3	1.294 (2)	O2—H1	0.8745
			
N5—C1—C6	117.59 (15)	H6A—C6—H6B	109.5
N5—C1—C2	123.80 (14)	C1—C6—H6C	109.5
C6—C1—C2	118.61 (14)	H6A—C6—H6C	109.5
C3—C2—C1	111.08 (13)	H6B—C6—H6C	109.5
C3—C2—H2A	109.4	N1—C7—H7A	109.5
C1—C2—H2A	109.4	N1—C7—H7B	109.5
C3—C2—H2B	109.4	H7A—C7—H7B	109.5
C1—C2—H2B	109.4	N1—C7—H7C	109.5
H2A—C2—H2B	108.0	H7A—C7—H7C	109.5
O1—C3—N1	121.38 (17)	H7B—C7—H7C	109.5
O1—C3—C2	122.69 (16)	C3—N1—C4	122.68 (14)
N1—C3—C2	115.92 (14)	C3—N1—C7	119.17 (15)
N2—C4—N4	110.65 (13)	C4—N1—C7	117.80 (14)
N2—C4—N1	125.12 (14)	C4—N2—N3	106.54 (13)
N4—C4—N1	124.04 (13)	C5—N3—N2	107.42 (13)
N3—C5—N4	110.76 (15)	C5—N4—C4	104.61 (13)
N3—C5—H5	124.6	C5—N4—N5	123.08 (13)
N4—C5—H5	124.6	C4—N4—N5	131.27 (12)
C1—C6—H6A	109.5	C1—N5—N4	115.05 (13)
C1—C6—H6B	109.5		

### Hydrogen-bond geometry (Å, º)

**Table:**

*D*—H···*A*	*D*—H	H···*A*	*D*···*A*	*D*—H···*A*
C2—H2*A*···N3^i^	0.97	2.58	3.449 (3)	149
C5—H5···O1^ii^	0.93	2.29	3.211 (2)	173
O2—H1···N3^iii^	0.87	2.08	2.939 (2)	167

Symmetry codes: (i) −*x*+1, −*y*+1, −*z*; (ii) *x*+1/2, *y*+1/2, *z*; (iii) −*x*+1, *y*, −*z*−1/2.
