# Supplementary figures and images for: Crystal structure of 6,9-dimethyl-7H-[1,2,4]triazolo[4,3-b][1,2,4]triazepin-8(9H)-one 0.40-hydrate
Source: Acta Crystallogr E Crystallogr Commun. 2015 Jan 1;71(Pt 1):o1–2. doi: 10.1107/S2056989014025687 (PMC4331897; doi:10.1107/S2056989014025687)

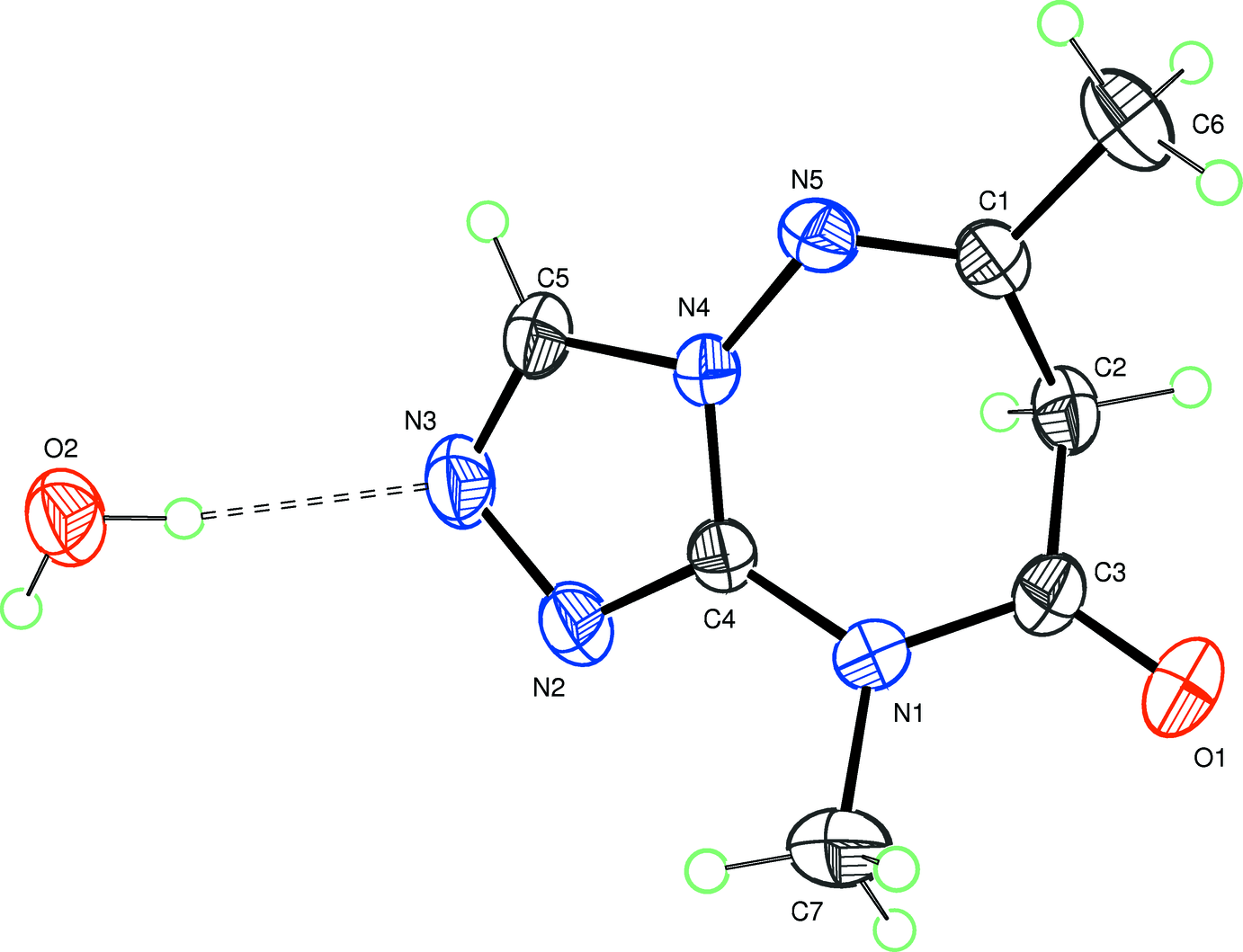

Supplement: Supplementary file 4 [file e-71-000o1-fig1.tif]

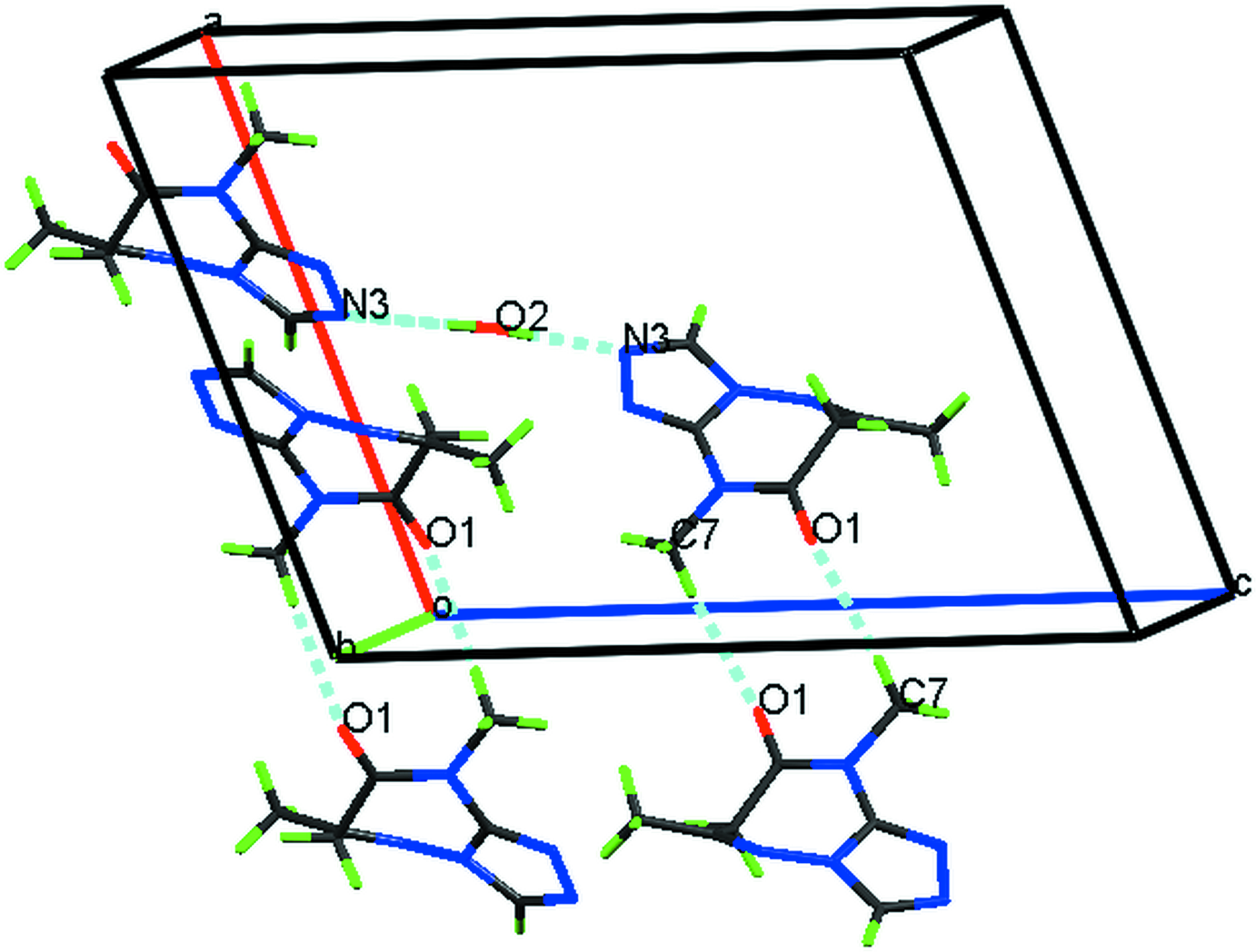

Supplement: Supplementary file 5 [file e-71-000o1-fig2.tif]
